# Supplementary material for: MetaRibo-Seq measures translation in microbiomes
Source: Nat Commun. 2020 Jun 29;11:3268. doi: 10.1038/s41467-020-17081-z (PMC7324362; doi:10.1038/s41467-020-17081-z)
Supplement: Supplementary file 10 — Supplementary Data 7 [file 41467_2020_17081_MOESM10_ESM.zip › File2/Confidence_High_Taxonomy/115689_out.krona.html]

Javascript must be enabled to view this page.

members
magnitude
magnitudeUnassigned
count
unassigned
taxon
rank

115689\_out

3

2
superkingdom
3

976
3
phylum

class
3
200643

171549
order
3

171552
2
family

838
genus
2

species

SRS016815\_contig\_number\_contig-100\_178.201892
1
1433844

60133
1

SRS019607\_contig\_number\_25441
species

1
family
815

1
genus
816

species

SRS049959\_contig\_number\_45789
1
1262751
